# The Sports-Related Injuries and Illnesses in Paralympic Sport Study (SRIIPSS): a study protocol for a prospective longitudinal study

**DOI:** 10.1186/s13102-016-0053-x

**Published:** 2016-08-30

**Authors:** Kristina Fagher, Jenny Jacobsson, Toomas Timpka, Örjan Dahlström, Jan Lexell

**Affiliations:** 1 Department of Health Sciences, Rehabilitation Medicine Research Group, Lund University, PO Box 157, 221 00 Lund, Sweden; 2 Department of Medical and Health Sciences, Athletics Research Center, Linköping University, 581 83 Linköping, Sweden; 3 Department of Behavioural Sciences and Learning, Linköping University, 581 83 Linköping, Sweden; 4 Department of Neurology and Rehabilitation Medicine, Skåne University Hospital, 221 85 Lund, Sweden; 5 Department of Health Science, Luleå University of Technology, 971 87 Luleå, Sweden

**Keywords:** Athletic injuries, Epidemiology, Research protocol, Sports for persons with disabilities, Sports medicine

## Abstract

**Background:**

Paralympic sport provides sporting opportunities for athletes with a disability, with the Paralympic Games as the main event. Participation in sport is, however, associated with a significant risk for sustaining injuries and illnesses. Our knowledge of sports-related injuries and illnesses in Paralympic sport is very limited and there are no large-scale epidemiological cohort studies. The purpose here is to present a protocol for a prospective longitudinal study: The Sports-Related Injuries and Illnesses in Paralympic Sport Study (SRIIPSS).

**Methods/design:**

An argument-based method for investigation of design problems was used to structure the study protocol. The primary requirement of the protocol is to allow prospective studies over time and include exposure to both training and competition. To reflect the complexity of Paralympic sport with athletes’ pre-existing impairments, use of assistive equipment, pain and other and medical issues, it is required that the data collection system is specifically adapted to Paralympic sport. To allow the collection of data, at the same time as there is limited access to coaches and medical personnel, it is advantageous that data can be collected online directly from the athletes. Based on this a self-report athlete monitoring system will be developed, where the athletes can enter data weekly via their mobile phones or lap-tops. Data will be collected from around 100 Swedish Paralympic athletes for approximately 1 year, which will allow us to i) prospectively estimate the annual incidence of sports-related injuries and illnesses and ii) explore risk factors and mechanisms for sustaining sports-related injuries and illnesses based on athlete exposure and training loads.

**Discussion:**

For effective implementation of injury and illness prevention measures, comprehensive epidemiological knowledge is required. This study will be the first prospective longitudinal self-report study of sports-related injuries and illnesses in Paralympic sport over a longer period of time. The results will eventually contribute to the development of evidence-based preventive measures specifically adapted to Paralympic sport in order to provide safe and healthy sport participation. Thereby, the project will be of relevance for Paralympic athletes at all levels and to the Paralympic Movement.

**Trial registration:**

The study is registered at ClinicalTrials.gov (Identifier: NCT02788500; Registration date: 22 May 2016).

## Background

Sport for athletes with disabilities has existed for more than 100 years. Today, the global network ‘The Paralympic Movement’ provides sporting opportunities for Paralympic athletes with physical, visual or intellectual impairments, from grassroots to elite level, with the Paralympic Games as the main event. During the past decades, Paralympic sport has seen a large development in both the number of athletes, sports performance and technology [1], and many Paralympic athletes have reached performances similar to able-bodied elite athletes [2].

It is well-known that physical activity and participation in sport generates several positive health effects [3, 4]. Low physical fitness and reduced physical activity is associated with many adverse health events, including major non-communicable diseases [5]. Participation in sport is therefore of great importance, especially for persons with disabilities, as individuals with a chronic disease or disability have lower physical fitness compared to non-disabled individuals [6]. Sport is today included in most rehabilitation programs for people with disabilities, to promote both physical and psychological well-being [7, 8].

### Injuries and illnesses in Paralympic sport

Participation in sport is, however, associated with a significant risk for sustaining injuries and illnesses that may have long-lasting effects, including mortality, morbidity and high costs for society [9, 10]. Remaining free of injury and illness has therefore become a fundamental component of successful performance in sport [11].

Previous research has shown that injury rates in Paralympic sport are generally high with a trend towards more injuries compared with sport for able-bodied athletes [12]. Injury patterns related to the impairment, the equipment involved and the specific mechanics of the sport have been proposed to be related to the injuries [13]. Maintaining health in athletes with already existing disabilities can be problematic. The athletes may have complex pre-existing medical conditions, such as neurodegenerative disorders, spinal cord injury, amputations, rare syndromes with anomalies in different body systems, vision loss and intellectual impairments, and medical issues like autonomic dysreflexia, infections, hyperthermia, skin lesions, spasticity, fatigue, pain and epilepsy can be present [13]. Moreover, the athletes may be exposed to repetitive and sometimes improper biomechanical load in their daily life [14, 15]. Based on the facts that the Paralympic Games is now one of the world’s largest multi-sport events [1] and that training intensity and performance levels have increased during the past years [16], there are surprisingly few epidemiological studies covering sports-related injuries and illnesses in Paralympic sport. Thus, further studies are needed to ensure the development of safe participation in Paralympic sport.

### Sports injury research

Recent research has shown that several categories of sports-related injuries are preventable [17–19]. However, for effective implementation of injury prevention measures, comprehensive epidemiological knowledge is required [9, 20]. To reduce overtraining, injuries and illnesses, regular monitoring of athletes is an important aspect in athletic preparation [21, 22]. Although the International Paralympic Committee (IPC) has successfully implemented an epidemiological surveillance system during the Paralympic Games [23], there is still a lack of longitudinal prospective data following Paralympic athletes over entire training seasons [12]. A recent review identified large differences in injuries across sports and highlighted the need for sport-specific studies [24]. Current studies within Paralympic sport vary in quality and have mainly recorded injuries related to trauma, medical attention or time loss. Most studies are retrospective and have only recorded injuries during competitions. In addition, a diversity of injury definitions have been used and most studies have not examined impairment-related risk factors and injury severity [12, 25]. Thus, there is a need for further longitudinal epidemiological studies that prospectively assess sports-related injuries and illnesses in Paralympic sport based on risk exposure.

Today, most injury surveillance systems exist in professional and commercial able-bodied elite sport settings [26], for example soccer, tennis and rugby [27–29]. In addition, many of the surveillance systems require that medical practitioners complete the injury report form [23, 28–30]. However, the characteristics, preconditions and contexts differ between sports [31, 32]. For example, medical attention injuries may be difficult to apply when there is limited access to medical personnel [33]. It has also been proposed that some methods for injury registration may underestimate overuse injuries [34]. Also, in terms of injury capture rates, medical staff may underestimate the injury burden compared to athletes themselves [32].

### Athlete monitoring in Paralympic sport

In Paralympic sport everyday access to coaches and medical personnel is scarce [15] and sport, especially in the Scandinavian countries, is primarily based on voluntary dependency [35]. Moreover, Paralympic sport has a wide geographical spread, both in Sweden and internationally, and involves more than 28 different sports and 10 different impairments types [1] (Table 1). In addition, patterns of sports-related injuries and illnesses differ in some ways from those among able-bodied athletes, as the impairment itself is involved in the cause and consequential chains [15]. The impairment that the athlete has may also cause difficulties in the definition and interpretation of sports-related injuries and illnesses. However, health should not only be related to the absence of a disease or an injury. It also includes the individuals’ capacity to carry out activities in relation to their self-perceived functioning and health [36].

**Table 1:** Eligible impairment types and sports in The Sports-Related Injuries and Illnesses in Paralympic Sport Study (SRIIPSS)

Impairments	Summer sports		Winter sports
Impaired muscle power Impaired passive range of movement	Archery	Athletics	Alpine Skiing
	Boccia	Canoe	Biathlon
	Cycling	Equestrian	Cross Country Skiing
Limb deficiency	Football-5-a-side	Football-7-a-side	Ice Sledge Hockey
Leg length difference	Goalball	Judo	Snowboard
Short stature	Powerlifting	Rowing	Wheelchair Curling
Hypertonia	Sailing	Shooting	
Ataxia	Sitting volleyball	Swimming	
Athetosis	Table tennis	Triathlon	
Vision impairment	Wheelchair basketball	Wheelchair fencing	
Intellectual impairment	Wheelchair rugby	Wheelchair tennis	

Today, there is growing evidence that self-report measures are sensitive and reliable tools to monitor athletes’ health [22, 37, 38]. For example, Jacobsson and co-workers [33] reported the development of a web-based self-report system for the Swedish Athletics study. They found that to allow specific analyses of overuse injuries, recording of partial time-loss injuries and regular collection of self-reported data over time was necessary in order to find searches for complex aetiological patterns. It is well-known that many injury-related musculoskeletal incidents result from the cumulative effects of smaller amplitude of micro-traumatic forces developing over time [39]. Therefore, to prevent injuries and overuse problems to develop into chronic conditions, it is useful to have self-report data that highlights the small and early decrements in functioning [40]. Also, data on other medical conditions, such as illnesses, training availability and training load, are important to allow us to understand health conditions beyond injuries [41, 42]. For example, loss of training time due to a health problem has recently been proposed to be a major determinant of success and failure [42]. Recent research in able-bodied athletes also indicates that a high chronic workload seems to decrease the risk of injuries, whereas excessive and rapid increases in training loads are likely to cause a large proportion of injuries [43].

With a systematic longitudinal self-report surveillance system based on exposure it would be possible to observe trends and risk factors of sports-related injuries and illnesses in Paralympic sport and thereby have a basis for the development of specific preventive measures. However, for injury surveillance data to be useful for prevention, a theoretical framework is required to understand how factors representative of the target population influence injuries and illnesses [44]. Moreover, to improve reach, implementation and maintenance it has been recommended that surveillance systems are user friendly and accessible in a wide range of form [45], in this case adapted for persons with physical, visual or intellectual impairments. To capture all sports-related injuries and illnesses in Paralympic sport, to obtain valid data and to allow specific injury prevention measures, there is a need for injury surveillance systems to be specifically tailored to Paralympic sport, targeting a wide range of para-athletes.

## Purpose

The purpose here is to present a protocol for a prospective longitudinal study: The Sports-Related Injuries and Illnesses in Paralympic Sport Study (SRIIPSS). In this study we will develop a web-based system that allows self-report data to be collected from Paralympic athletes. Based on these data, we will subsequently: i) record and prospectively estimate the annual incidence of sports-related injuries and illnesses among Swedish Paralympic athletes and ii) explore risk factors and mechanisms for sustaining sports-related injuries and illnesses based on athlete exposure and training loads.

## Methods

### Study design and rationale

The SRIIPSS is an epidemiological cohort study aimed to prospectively collect self-report data on the incidence and risk exposure of sports-related injuries and illnesses during training and competition during approximately 1 year among Swedish Paralympic athletes. The study will follow the STROBE (Strengthening the Reporting of Observational studies in Epidemiology) guidelines [46] and is registered at ClinicalTrials.gov (Identifier: NCT02788500; Registration date: 22 May 2016).

**Fig. 1:**
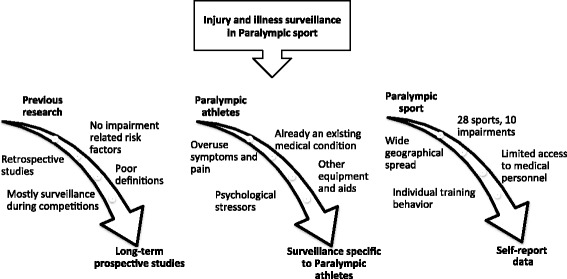
Requirements and associations underlying the study protocol for The Sports-Related Injuries and Illnesses in Paralympic Sport Study (SRIIPSS)

The research team, comprising sports injury epidemiologists, physicians and physical therapists, used an argument-based method for investigation of complex design problems to structure the study protocol. In this operational research process, an interdisciplinary approach is used to find logical conclusions, test formal soundness, and thereafter establish a design rationale [47, 48]. The focus of the design rationale is to document both the development process and resulting design [48]. The argumentation included discussions about the types of data that will be collected, data storage, and ethical and logistical considerations. Examination of requirements was followed by iterated drafting of protocol specifications based on previous research [12, 13, 24, 25], the athletes’ own perceptions of sports-related injuries and illnesses [15] and the context of Swedish Paralympic sport [49] (Fig. 1).

The primary requirement of a protocol for longitudinal epidemiological studies in Paralympic sport is to allow prospective studies that cover at least an entire season and include exposure to both training and competition. Second, to reflect the complexity of Paralympic sport, for example pre-existing impairments, use of assistive equipment, pain and other and medical issues, it is required that the design of a surveillance system is specifically adapted to Paralympic sport. Third, to allow the collection of epidemiological data and individual training behaviors, at the same time as there is limited access to coaches and medical personnel, it is advantageous that longitudinal data can be collected online directly from the athletes. There is also increasing evidence that psychological and behavioral factors contribute to the process leading to several sports injuries [50, 51]. Therefore, a psychological profile will be added to identify factors and behaviors related to pain and other activity-limiting sensations [50].

### Setting and participants

Potential participants will be recruited through the Swedish Paralympic Program, which covers candidates for the Summer and Winter Paralympic Games. The total number of athletes in the Swedish Paralympic program is around 100. All potential participants will be invited by post or email. They will receive information about the proposed study, a request for a contact e-mail and phone number, a consent form for participation and a prepaid return envelope. To be eligible for the study the athlete should belong to one of the ten eligible impairment types, according to the IPC classification system, and participate in either a Paralympic summer or winter sport (Table 1). The following inclusion criteria will be used: age 18–55 years, being a registered athlete within the Swedish Paralympic Program, being able to communicate in Swedish and having the opportunity to respond weekly to a web-based questionnaire. As the number of Swedish Paralympic athletes is small and this is an understudied topic, a total population design will be applied.

### Protocol implementation

The SRIIPSS will employ approaches similar to those that have been used in previous sports injury research [9, 23, 30, 33, 52]. It is, however, hypothesized that sports-related injuries and illnesses among Paralympic athletes differ from sport for able-bodied athletes [13, 15]. Existing research has shown that the accessibility, compatibility, interface and design of questions influence the outcome of athletes’ self-report measures [38]. Therefore, we will specifically adapt the protocol to Paralympic sport and thereafter implement the data collection.

For the data recording a commercial product for collection of survey data online (Briteback AB, Norrköping, Sweden; www.briteback.com) will be used. The research team participates in the development of the system to specifically adapt it to, for example, visually impaired athletes. The product enables definition of personal usernames and passwords to protect data from unauthorised use.

A pilot study is planned where we will enroll approximately twenty athletes with different impairments through a convenience sample from the Swedish Paralympic Program. The pilot study aims to evaluate the feasibility of the data collection system, to assure that it works for athletes with physical, visual and intellectual impairments, and to recognize and solve potential problems. The athletes will be asked to fill in the survey during 4 weeks. The pilot study period will end with a cognitive walkthrough to review the primary protocol and also validate the data. This is an evaluation method that aims to assess usability problems of a new tool early in the design process [53]. The athletes and researchers will be asked to evaluate and provide solutions for the proposed system.

Data will be collected from the end of 2016 and continue for approximately 1 year. At the start of the study the athletes fill in baseline information and a psychological profile. The athletes will thereafter be asked to report sports-related injuries and illnesses weekly. The primary outcome of the study is the incidence of injuries and illnesses, divided into a sports-related injury or illness. All data will be based on sport exposure and analyzed for the mechanism of sports-related injuries and illnesses. A closure form will be used when the athlete is back in full training following an injury or illness (Table 2).

**Table 2:** Overview of the outcome measures of the SRIIPSS

Outcome measure	Parameter	Measurement
Baseline data	Gender, age, impairment, sport, training hours, previous injury/illness, medication, aids, anthropometrics, pain	SRIIPSS protocol for baseline data
Psychological profile	Perceived motivational climate	Perceived Motivational Climate in Sport Questionnaire (PMCSQ)
	Active coping	BRIEF COPE
	Body consciousness and body competence	Body Consciousness Questionnaire Scale (BCQ-s)
	Hyperactivity	Diagnostic and Statistical Manual of Mental Disorders (DSM-IV)
	Psychological commitment to exercise	Commitment to Exercise Scale (CTeS)
	General well-being	Likert-scale
Weekly athlete report	Injuries, illnesses, pain, psychological stressors, amount of training and competition, training intensity, sleep	SRIIPSS protocol for weekly athlete data Likert-scale
Injury report	Onset of injury, mechanism of injury, present symptoms, type of injury, impairment-related factors, medical contacts	SRIIPSS protocol for weekly injury data
Illness report	Onset of illness, present symptoms, type of illness, impairment-related factors, medical contacts	SRIIPSS protocol for weekly illness data
Injury/illness closure form	Diagnosis of injury/illness, severity of injury, treatment, perceived risk factors	SRIIPSS protocol for injury/illness closure data

### Definitions of injury and illness

The main injury definition in the SRIIPSS follows, with some alterations, the definitions previously used for soccer, rugby union, the Olympic Games and athletics [27, 29, 30, 52]:

*Any new musculoskeletal pain, feeling or injury that result from participation in Paralympic sport (training or competition) and cause changes in normal training/competition to the mode, duration, intensity or frequency, regardless of whether or not time is lost from training or competition.*

An illness including psychological complaints will be defined as:

*Any new illness or psychological complaint that cause changes in normal training/competition to the mode, duration, intensity or frequency, regardless of whether or not time is lost from training or competition.*

The reported incidents will be categorized as ‘sudden onset’ or ‘gradual onset’. A sudden onset incident refers to an incident caused by a specific identifiable episode resulting in a rapid onset of experienced distress. Subsequently, sudden onset injuries will be categorized according to the cause of the incident as: i) traumatic injuries – a condition caused by an identifiable single external transfer of energy (for example, a bone fracture caused by a fall or a ligament tear caused by contact with an obstacle), or ii) overuse injuries – a condition to which no identifiable single external transfer of energy can be associated (examples of overuse sudden onset injuries include tendon tears).

A gradual onset incident refers to a condition that manifests itself over a period of time, or when there is a gradual increase in the intensity of experienced distress, without a single identifiable event being responsible for the condition. Examples of gradual onset conditions include overtraining syndromes and overuse injuries, such as tendinosis and tendinopathies [52].

The arguments for using these definitions are based, first, on that the incidents will be recorded from the athlete’s own subjective perspective, feelings and experiences of pain, injury and illness. Second, these definitions support the concept of partial time-loss injury [33, 54]. In order to capture these injury events it is important to identify complex background patterns for overuse injuries [33, 34]. Third, this athlete population already has an existing impairment and is exposed to elements not present in abled-bodied athletes. To allow for a better understanding of sports-related injuries and illnesses in this population, we believe that it is important to assess various factors such as pain, injuries, illnesses, and psychological stressors in order to obtain a comprehensive picture of Paralympic athletes’ health. To allow comparisons with previous studies of Paralympic athletes and non-disabled athletes, the incidents will at a secondary level of the analysis be identified by time-loss and tissue damage.

In agreement with previous injury surveillance studies [28, 52] a recurrent condition will be defined as:

*An incident of the same type and at the same site linked to an index incident and which occurs after an athlete’s return to full function and participation (“full recovery”) from the index recordable incident.*

The incidents will be subcategorized into re-injuries and exacerbations. Categorization of subsequent injuries – new, recurrent, exacerbation or multiple – will be taken into account using the Subsequent Injury Categorization (SIC) model before analysis [55].

### Outcome measures

#### Baseline data

Baseline data will be collected using a web-based questionnaire including: i) participant characteristics (i.e., gender, age, height and weight); ii) demographic data; iii) impairment characteristics (i.e., type of impairment, use of assistant devises, medications); and iv) sport characteristics (i.e., type of sport, preconditions, hours per week involved in training and competition). The athletes will also be asked about existing injuries, illnesses and pain. In addition, women will be asked about menstruation and use of contraceptives.

### Psychological profile

Data for a psychological profile will be collected at baseline and at the end-point of the study based on the affective adaptation framework [56] and the psychological flexibility model [57]. These models are based on behavioral risk factors including awareness and explanatory processes of sensory information, affective reactions, enduring psychological factors and maladaptive thoughts and capacity to change behavior.

Measurement of body consciousness and body competence will be based on the Body Consciousness Scale (BCS) [58]; six items from the hyperactivity definition in the Diagnostic and Statistical Manual of Mental Disorders (DSM-IV) were added. The Brief Cope instrument (Kristiansen 2008) will be used to measure active coping including planning, mood, acceptance and maladaptive behavior. The Perceived Motivational Climate in Sport Questionnaire (PMCSQ) will be used to assess perceived demands of the social sporting environment including performance orientations and mastery/task accomplishment [59]. The individual’s psychological commitment to the activity of exercising and tendencies to rigidity in training will be measured by the Commitment to Exercise Scale (CtES) [60]. All psychological measures have shown satisfactory measurement properties when used in sports medicine research [50].

### Athlete weekly e-diary

To collect data on new sports-related injuries and illnesses as well as on exposure, the athletes will be asked to fill in a weekly e-dairy based on one developed by Jacobsson et al. [33] and adapted for Paralympic sport. Each week, an alert is automatically sent to the participants’ email address and mobile phone, asking them to fill in the weekly questionnaire about their current health status (injuries, illnesses and pain), the amount of training, intensity of training, whether the training is performed at full capacity, competitions, sleep, general well-being and medical contacts. In addition, anxiety/depression and pain/discomfort will be evaluated using two questions from EQ-5D [61].

If the athlete reports a new injury or illness in their weekly e-dairy, a link to an injury or illness report form will be provided, where additional information on the incident are to be reported. The study coordinator (KF) monitors the reported data. If a participant is absent from training because of an undiagnosed injury or illness lasting longer than 3 weeks, the participant will be contacted by phone or email. The athletes will be asked to go through an examination by a sports physician or sports physiotherapist to confirm the clinical diagnosis, in order to validate the data.

Internal training loads will be recorded based on a modified version from Gabbett & Jenkins [62] and Foster et al. [63]. The athletes will be asked to estimate their total rating of perceived exertion (RPE) during the past week on a 0–10 point RPE-scale [62]. Previous research has demonstrated that the RPE method is a valid method to quantify exercise training during a wide variety of exercise types [63], and the method has also been shown to provide valid estimates when compared to heart rate and blood lactate concentration. The training load will be calculated by multiplying the training session intensity with the duration of the training during the week and will be further analysed with the relationship of injury/illness, acute loads and chronic loads [43, 62, 64].

Assistance will be provided, if necessary, for athletes with vision or intellectual impairment, or severe physical impairment. At the start of the study the athletes will be educated about the importance of monitoring. Feedback will be given, if desired, to allow data to be used to the athletes’ benefit.

### Injury report form

The injury report form is a modified version of the form described by Jacobsson et al. [33], originally based on the soccer consensus by Fuller et al [27] and the International Olympic Committee (IOC) groups [30]. This injury report system has been shown to be feasible for self-report data in other individual sports, such as athletics [31]. The athletes will be asked about the onset of injury, mechanism of injury, contributing factors, anatomical site and recurrence of earlier injury. The athletes will also be asked about presenting symptom(s) or sign(s) and suspected aetiology (a list of common categories of causes is provided). The system is further adapted to be applicable to athletes with impairments. For example, questions will be added regarding the impairment and equipment used.

### Illness report form

Illnesses will be recorded based on a combination of data collection procedures for epidemiological studies in athletics and during the Paralympic Games [52, 65]. The athletes will be asked about presenting symptom(s) or sign(s), affected system, mode of onset and suspected aetiology (a list of common categories of causes is provided). The report form is further adjusted to be applicable to athletes with impairments.

### Injury closure form

When the athletes report that they are back in normal training following an injury, they will be asked to fill in an injury closure form with: i) time off full training; ii) final diagnosis; iii) person who made the diagnosis; iv) treatment(s) received; and v) perceived risk factors of injury including impairment related factors. Additionally, there will be the possibility to provide personal comments about the incident reported. The assessment of severity starts on the following day, if the athlete is unable to take part in full and/or normal training and/or competition. Severity will be reported as days absent from training: minor (1–3 days); mild (4–7 days); moderately serious (8–28 days); serious (>28 days-3 months); long-term (3–6 months); and catastrophic injuries [9].

Finally, to describe the patterns of injury, a matrix adjusted to Paralympic sport will be used to categorize the coded injury data according to injury type (nature of injury) and anatomic location (body region). This is based on the Barell Injury Diagnosis Matrix [66] and modified for overuse injuries by Hauret et al. [39] and Jacobsson et el. [31]. The incidents will be classified by mode of onset and will be further analyzed by athlete classification and specific impairment type. A group consisting of two physiotherapists and two physicians with a background in sports medicine and Paralympic sport will classify each self-reported diagnosis of sports-related injuries and illnesses into a diagnostic code according to The International Classification of Diseases (ICD). To confirm the diagnosis of fracture/stress fracture, an x-ray will be required.

### Statistical analysis plan

The primary variables for the descriptive analyses are records of the incidence of sports-related injuries and illnesses, i.e., recordings of injury or illness events that are conditioned on that the participant is ‘healthy’ at the start of data collection period. Incidences will be calculated separately for injuries and illnesses. The incidence rates will be calculated as the number of new incidents divided by total athlete exposure hours (per 1000 h of sport participation). Health-incidents not related to sport will be analyzed separately. All events will be evaluated according to the mechanism of sports-related injuries and illnesses (independent variables). The data will be assessed for normality and will be presented using quantitative descriptive statistics (mean, median, standard deviation, minimum and maximum for continuous data and frequency and proportion (%) for categorical data). Differences in proportions between different categories (e.g., age-groups, gender, impairments, previous injury, competition frequency and level of severity of impairment) will be analyzed as covariates using chi-square statistics. The primary end point for the injury risk analyses will be time to injury. The incidence data will be further analyzed with the Kaplan-Meier survival analysis method, the log-rank test and Cox proportional hazards regression. To analyze the relationship between training loads and injury and illness incidence, Pearson product moment correlation coefficients will be used. A significance level of 0.05 and 95 % confidence intervals will be used in all tests. The data will be analyzed using IBM SPSS Statistics version 23.

## Discussion

We here present a protocol for a prospective epidemiological study of sports-related injuries and illnesses in Paralympic sport and provide arguments related to its design. To the best of our knowledge, no study has prospectively and comprehensively assessed the epidemiology of sports-related injuries and illnesses in Paralympic sport over a longer period of time. Health related problems, in particular musculoskeletal injuries, are common in sport [10]. Previous studies have indicated that sports-related injuries and illnesses is a major concern also within Paralympic sport [23, 24]. Moreover, few studies have analyzed the onset and diagnosis of sports-related injuries and illnesses and further analyzed impairment-related and sport specific risk factors. The proposed study protocol will be used to assess injures and illnesses over time and between different sports and populations. With sports-related injuries and illnesses incident cause data based on athlete exposure it will be possible to observe trends, potential interactions and risk factors over time, and thereby target sports with a higher risk.

It could be argued that a limitation is that no data on clinical examinations are included at baseline. However, self-report systems have previously been shown to enable valid and reliable recordings of sports-related incidents [67] and to monitor changes in athletes’ well-being [68]. This longitudinal research project covers a complex and understudied field. To move forward, basic and feasible research is required. In particular, long-term analyses of possible cause-relationships of sports-related injuries and illnesses by athlete classification and specific impairment type are of importance, as it is hypothesized that injuries and illnesses may be specific for athletes’ sport and impairment type [14]. Other challenges that long-term self-report studies may face are drop-out of participants, low response rates, and problems understanding and interpreting the questions. Moreover, this is the first study among Paralympic athletes with various physical, visual, and intellectual impairments, which requires that the proposed system work for all types of impairments.

The SRIIPSS is expected to lead to an in-depth understanding of the epidemiology of sports-related injuries and illnesses. The results will eventually contribute to the development of evidence-based preventive measures specifically adapted to Paralympic sport in order to provide safe and healthy sport participation. Thereby, the project will be of relevance for Paralympic athletes at all levels and to the Paralympic Movement.

## Abbreviations

BCS: Body consciousness scale
CtESm: Commitment to exercise scale
DSM-IV: Diagnostic and statistical manual of mental disorders
ICD: The International Classification of Diseases
IOC: International Olympic Committee
IPC: International Paralympic Committee
PMCSQ: The Perceived Motivational Climate in Sport Questionnaire
RPE: Rating of perceived exertion
SIC: Subsequent injury categorization
SRIIPSS: The Sports-Related Injuries and Illnesses in Paralympic Sport Study
