# Erratum to: The Sports-Related Injuries and Illnesses in Paralympic Sport Study (SRIIPSS): a study protocol for a prospective longitudinal study

**DOI:** 10.1186/s13102-016-0055-8

**Published:** 2016-09-27

**Authors:** Kristina Fagher, Jenny Jacobsson, Toomas Timpka, Örjan Dahlström, Jan Lexell

**Affiliations:** 1 Department of Health Sciences, Rehabilitation Medicine Research Group, Lund University, PO Box 157221 00, Lund, Sweden; 2 Department of Medical and Health Sciences, Athletics Research Center, Linköping University, 581 83 Linköping, Sweden; 3 Department of Behavioural Sciences and Learning, Linköping University, 581 83 Linköping, Sweden; 4 Department of Neurology and Rehabilitation Medicine, Skåne University Hospital, 221 85 Lund, Sweden; 5 Department of Health Science, Luleå University of Technology, 971 87 Luleå, Sweden

## Erratum

After publication of the original article [1], it came to the authors’ attention that there was a formatting error within Table 1 which reduced the legibility of the content. In row 1 of the ‘Impairments’ column, “Impaired muscle power” and “Impaired passive range of movement” appear on the same line, as opposed to split over two lines within the same cell. This has been corrected in the original article and the correct version of Table 1 is published here.

**Table 1:** Eligible impairment types and sports in The Sports-Related Injuries and Illnesses in Paralympic Sport Study (SRIIPSS)

Impairments	Summer sports		Winter sports
Impaired muscle power Impaired passive range of movement	Archery	Athletics	Alpine Skiing
	Boccia	Canoe	Biathlon
	Cycling	Equestrian	Cross Country Skiing
Limb deficiency	Football-5-a-side	Football-7-a-side	Ice Sledge Hockey
Leg length difference	Goalball	Judo	Snowboard
Short stature	Powerlifting	Rowing	Wheelchair Curling
Hypertonia	Sailing	Shooting	
Ataxia	Sitting volleyball	Swimming	
Athetosis	Table tennis	Triathlon	
Vision impairment	Wheelchair basketball	Wheelchair fencing	
Intellectual impairment	Wheelchair rugby	Wheelchair tennis	
